# Geographic origin and migration phenology of European red admirals (*Vanessa atalanta*) as revealed by stable isotopes

**DOI:** 10.1186/s40462-018-0143-3

**Published:** 2018-12-21

**Authors:** Oskar Brattström, Anatoly Shapoval, Leonard I. Wassenaar, Keith A. Hobson, Susanne Åkesson

**Affiliations:** 1 Department of Biology, Center for Animal Movement Research, Lund University, Ecology Building, SE-223 62 Lund, Sweden; 2 Department of Zoology, University of Cambridge, Downing Street, Cambridge, CB2 3EJ UK; 3 Biological Station, Rybachy district, Kaliningrad, 238534 Russia; 4 Environment Canada, 11 Innovation Blvd, Saskatoon, Saskatchewan S7N 3H5 Canada; 5 Isotope Hydrology Section, International Atomic Energy Agency, A-1400 Vienna, Austria; 6 Department of Biology, Biological & Geological Sciences Building, Western University, London, Ontario N6A 5B7 Canada

**Keywords:** Geographic origin, Hydrogen isotopes, Insect migration, Migration phenology, Stable isotopes

## Abstract

**Background:**

Long-distance migration has evolved multiple times in different animal taxa. For insect migrants, the complete annual migration cycle covering several thousand kilometres, may be performed by several generations, each migrating part of the distance and reproducing. Different life-cycle stages and preferred orientation may thus, be found along the migration route. For migrating red admirals (*Vanessa atalanta*) it has been questioned if they reproduce in the most northern part of the range. Here we present migration phenology data from a two-year time series of migrating red admirals captured at Rybachy, Kaliningrad, in the northern part of Europe investigating time for migration, life-history stage (migration, reproduction) as well as site of origin in individual butterflies.

**Methods:**

Red admirals were captured daily at a coastal site during spring, summer and autumn in 2004 and 2005. For the sampled individuals, reproductive status and fuel content were estimated by visual inspection, and hydrogen isotopes (*δ*^2^H) were analysed in wing samples. *δ*^2^H values was compared with samples from two nearby reference sites in Estonia and Poland.

**Results:**

Analysis of hydrogen isotopes (*δ*^2^H) in red admiral wings showed that the spring cohort were of a southerly origin, while those caught in August or later in the autumn were from the local region or areas further to the north. All females caught during spring had developing eggs in their abdomen, but no eggs were found in late summer/autumn. There was a male-biased sex ratio during autumn and a difference in lipid content between years. When comparing the isotopic data with inland nearby locations, it was clear that the range of *δ*^2^H values (− 181 to − 78) was wider at Rybachy as compared to the two reference sites in Estonia and Poland (− 174 to − 100).

**Conclusions:**

During spring, migratory female red admirals arrived from the south and were ready to reproduce, while the autumn passage mainly engaged local and more northern individuals carrying large fuel deposits in preparation for long-distance migration. The phenology data suggest that individuals select to migrate in favourable weather conditions and that numbers may differ between years. Future studies should focus on individual sampling at a wide range of sites to reveal differential migration strategies and timing of migration between sexes and populations of migrating butterflies.

**Electronic supplementary material:**

The online version of this article (10.1186/s40462-018-0143-3) contains supplementary material, which is available to authorized users.

## Introduction

Animals have evolved different migration strategies, including repeated long-distance annual migration between sites used for breeding and wintering [4, 7], and multi-generational circannual migration in butterflies across wide latitudinal ranges [17, 48] to explore seasonal resources across the globe. Depending on resource availability, migration may be regular or irruptive and engage different numbers of individuals each year. Irruptive migration is regularly observed in birds and insects [23, 38].

The red admiral (*Vanessa atalanta*) performs a fairly regular migration in contrast to many of the long-distance migrating butterflies found in Europe, such as the painted lady (*Vanessa cardui*) (e.g. [19, 39, 48]) which shows larger variation between years. Migration varies in intensity, timing and origin [13, 15], but red admirals usually reach northern Europe in large numbers each spring. These immigrants reproduce and give rise to new generations that migrate south later in the season to reach areas suitable for reproduction during winter, although parts of the population apparently spend the winter hibernating. Traditionally red admirals were thought to overwinter in areas around the Mediterranean Sea in a state of reproductive diapause. However, modern studies have disputed this providing evidence that they are actively reproducing throughout winter and that the spring migration is performed by a new generation of adults [12, 14, 47]. Most reports of red admiral hibernation are from northern regions, but this behaviour seems to be of little importance for the new generation that hatches the following summer as there is no correlation between observed numbers overwintering and monitoring counts towards the end of the season [40]. It may however be possible that individuals from the eastern parts of the range are specialised winter hibernators even at more southerly latitudes [13]. Since most insect migrants are far more variable in their migration than, for example, birds (e.g. [7, 23]), we still lack long-term studies of insect migrants from the same location, and which may provide information on migration phenology, fuel load and reproductive status. Coastal locations are generally better suited than inland areas for observing red admirals (e.g. [10, 15, 28, 41]), because higher numbers often are a consequence of the funnelling effects of topography and the tendency of the red admirals to avoid crossing large water bodies [15].

The objective of this study was to obtain a continuous two-year time series of red admiral samples from one location to obtain detailed insights into the spring and autumn migration phenology, geographic origin, reproductive and migratory status of individual butterflies for this northern study site. Most other studies of migrating red admirals represent snap-shots in time (e.g. [30]; cf. [15]) or are from varied locations (e.g. [28]). Even though these studies have provided insights on the variable migration of the species, the daily pattern of migration and how migration intensity varies between years has not been documented systematically, with some exceptions [15]. We set up the following hypotheses: 1) we expected to catch red admirals of southern origin during spring migration, and from the north or local origin in autumn, 2) we expected no difference in sex ratio between seasons, 3) we expected a larger fraction of butterflies in spring to be prepared for reproduction, and 4) we expected no difference in fat content between seasons or years, as all individuals captured would be prepared for migration. To test these hypotheses we extensively sampled red admirals at one fixed coastal location at a bird ringing station at Rybachy, where large numbers of red admirals concentrate. Sampled butterflies were then analysed for stable hydrogen isotopes (*δ*^2^H) (to determine relative areas of natal origin), sex, breeding status, and lipid content.

## Materials and methods

### Study location and collection of red admirals

**Fig. 1:**
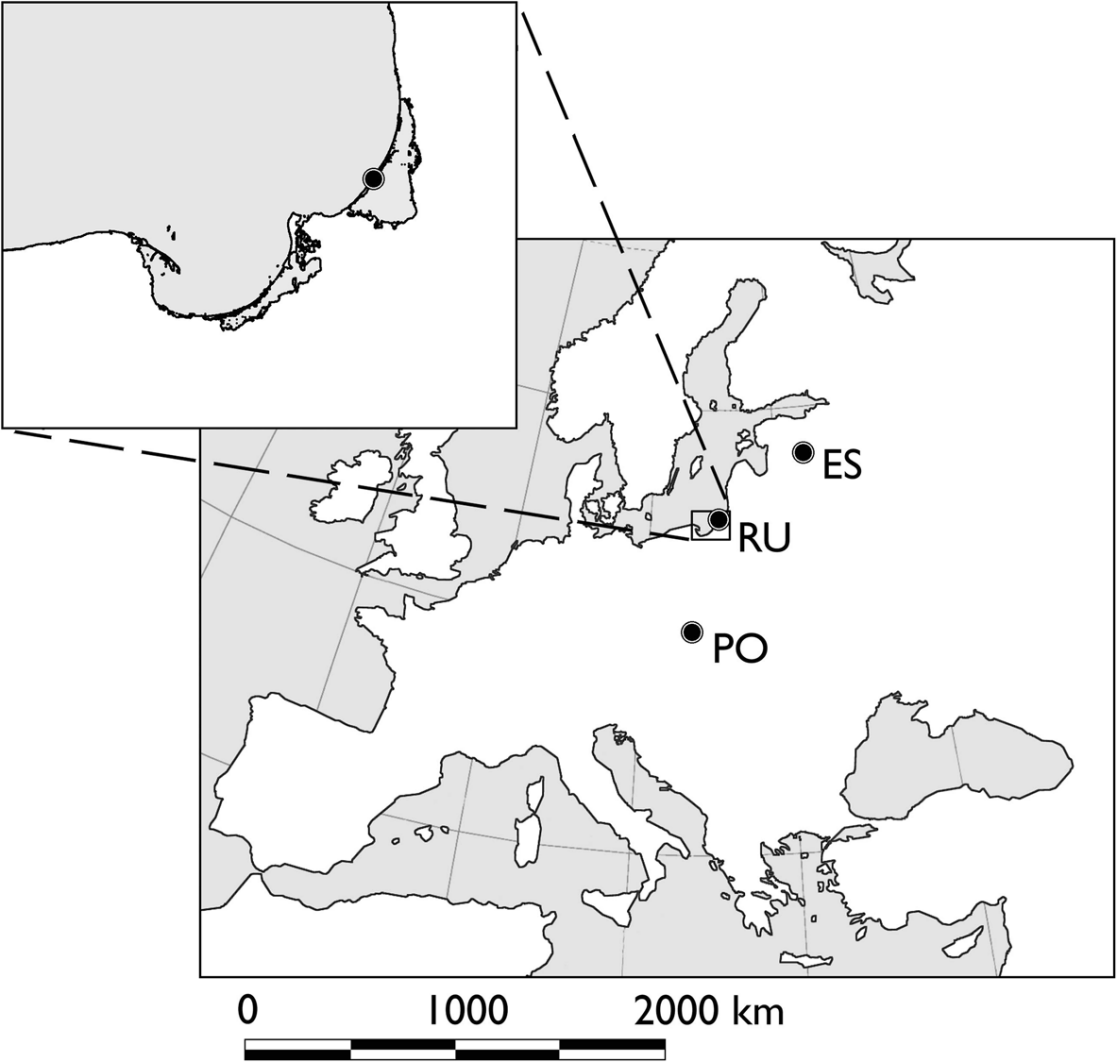
Location of sites were red admirals were captured from 2004 to 2005. Most red admirals in this study came from Rybachy in Kaliningrad in Russia (RU), located at the eastern shore of the Baltic Sea. Some additional material was also collected in Karilatsi in Estonia (ES), and in Czestochowa and Ktomnice in Poland (PO). Data from the inland capture sites in Estonia and Poland were compared with the coastal site at Rybachy

Rybachy (55°2’N, 12°8′E) is situated at the Curonian spit, a long and narrow peninsula at the eastern shore of the Baltic Sea (Fig. 1). Red admirals were collected in a large permanent trap of the “Rybachy-type”. This is a trap built from netting and shaped like a large funnel with an opening in the direction from which the migrants arrive, passively catching migrating birds and insects. At the far end of the trap is a collecting box. The dimensions of the trap are 70*35*15 m (Lengh*Width*Height) and it is operated daily between 1 April to 1 November on a yearly basis. Red admirals that were caught in the trap were euthanized using ethyl acetate and the wings were then removed for *δ*^2^H analyses. The wings were stored dry in glassine envelopes until isotopic analysis. The head and the body were stored in 99.9*%* ethanol for later analysis of sex, breeding status and lipid content. We captured red admirals over two spring and autumn seasons in 2004 and 2005. On days with large numbers of red admirals we sampled a fraction of the captured individuals (5–10). We recorded the numbers captured each day to be able to estimate yearly differences in the intensity and median date of migration.

### Analysis of hydrogen isotopes

We analysed hydrogen isotope ratios in the wings of red admirals compared our results of samples collected at our coastal site at Rybachy with two nearby inland sites in Estonia and Poland. These inland samples were represented by 15 individuals captured in Estonia (Karilatsi) between 11 and 28 August in 2004, and 20 individuals collected in 2005 between 14 September and 2 October at two locations in southern Poland (Czestochowa and Ktomnice) (Fig. 1).

All red admiral wing samples were soaked with chloroform-methanol solution (2:1) rinses, to remove surface oils that could affect the H isotope assays and air-dried. Stable hydrogen isotope analyses were conducted at Environment Canada in Saskatoon, using the comparative equilibration technique so that the values reported here are equivalent to non-exchangeable hydrogen [46, 52]. Stable-hydrogen isotope measurements of wings and the calibrated keratin standards were done on H_2_ derived from high-temperature (1300 °C) flash pyrolysis of wings and continuous-flow isotope-ratio mass spectrometry. The reference values used for our keratinous H standards calibration was − 197 ‰ and − 54 ‰ for CBS and KHS, respectively [46]. All H isotope results are expressed in the delta (*δ*^2^H) notation in units of per mil (‰), and normalised to the Vienna Standard Mean Ocean Water – Standard Light Antarctic Precipitation (VSMOW-SLAP) standard scale. Based on within-run measurements of a control standard (SPC) and consideration of within sample variance [53], the laboratory error for *δ*^2^H was <±2 ‰.

### Precipitation data

We acquired interpolated monthly *δ*^2^H values for the rainwater at Rybachy by using the Online Isotopes in Precipitation Calculator (OIPC) (http://wateriso.utah.edu/waterisotopes/index.html). Previous studies have shown that the *δ*^2^H values in butterfly wings are correlated to the *δ*^2^H of precipitation at their natal site (for details on the transfer function see, [14]). We used the OIPC data for precipitation to estimate the expected *δ*^2^H values for butterflies of local origin in the Rybachy region over the whole sampling period. Since the OIPC generates a mean *δ*^2^H value per month, we calculated a set of local mean *δ*^2^H for the middle third of each month and extrapolated the values in the first and last third of each month. This approach allowed us to assess whether captured red admirals were likely of local origin, or if they had migrated to the Rybachy area from the north (lower *δ*^2^H values than expected local values) or south (higher *δ*^2^H values than expected local values).

### Sex, breeding status and lipid class determination

**Fig. 2:**
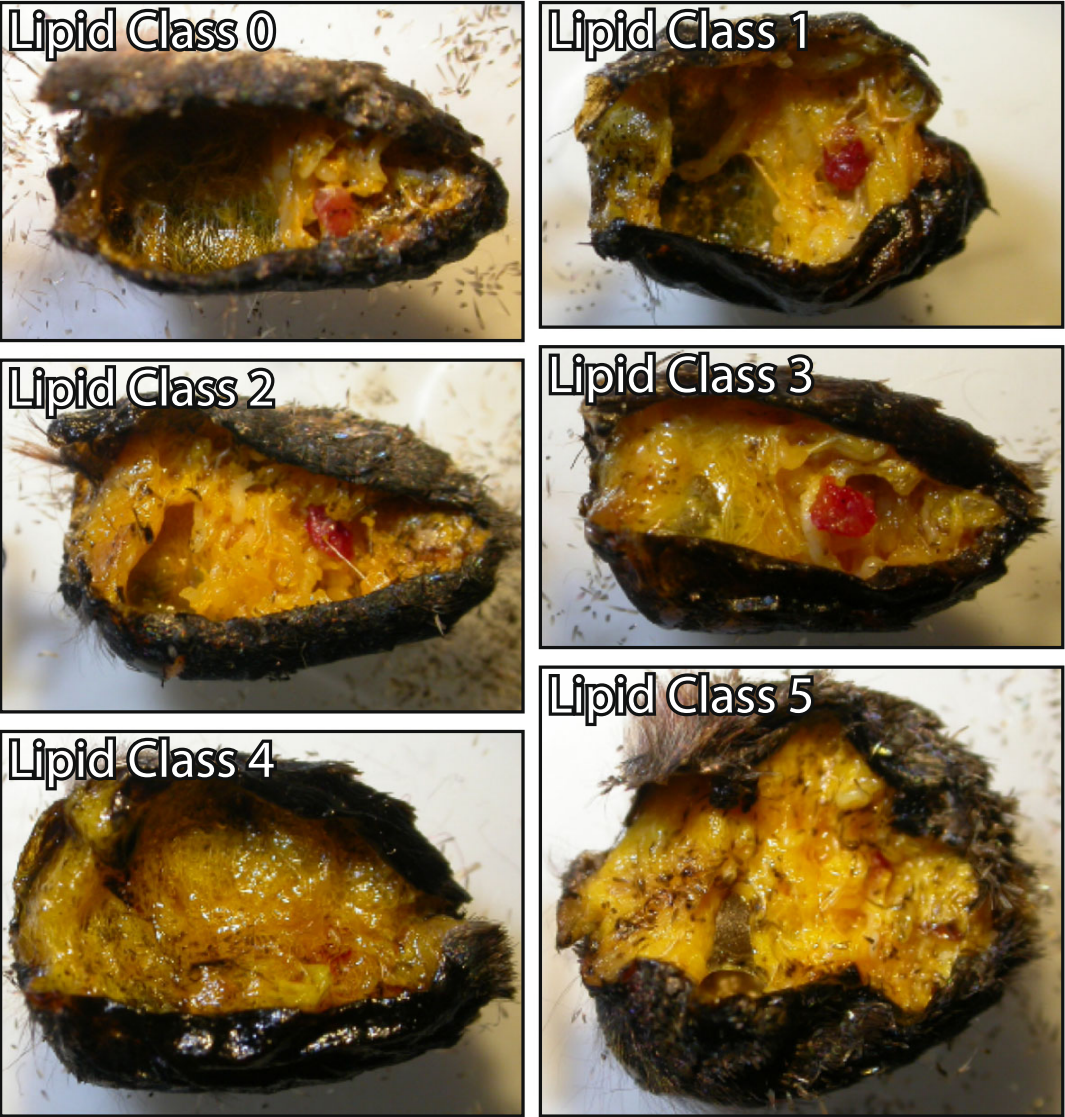
Visual scale for fat classification in red admirals (Lipid class 0-5). Descriptions for the fat classes are given in Table 1

To determine the sex of individual butterflies and evaluate the amount of lipid reserves, we dissected the abdomen of the red admirals captured at Rybachy. Sex was determined by visually inspecting the genitalia by microscope. To estimate lipid content we cut the wall of the abdomen open along one side using a fine pair of scissors and visually scored the amount of lipids in the abdominal cavity. When red admirals use up their lipid resources, a clear cavity begins to form in the stored abdominal fat. We estimated lipid reserves using a scale with six different classes from 0 (no fat) to 5 (abdomen cavity filled with fat). The criterion for each class is given in Table 1 and Fig. 2. In females, we also checked for the presence or absence of developing eggs to get an indication of breeding status.

**Table 1:** Criteria for division of samples from red admirals into different classes based on the abdominal lipid content. Proportion and number of individuals assigned to each class in the two years of the study is also presented. Images of one example from each class is presented as Fig. 2

Fat Class	Criteria for inclusion	*%* of total (*N*)	
		2004	2005
0	Abdominal compartment completely emptied of lipid	9*%* (11)	9*%* (8)
1	Large cavity in the abdominal lipid reserve	32*%* (38)	10*%* (9)
2	Small cavity in the abdominal lipid reserve	24*%* (28)	10*%* (9)
3	No visible cavity in lipid reserve, abdomen of normal size	31*%* (37)	25*%* (23)
4	Abdomen larger than normal because of substantial amounts of lipid, but not completely filled	1*%* (1)	17*%* (15)
5	Abdomen much larger than normal and completely filled with lipid	3*%* (3)	29*%* (26)

### Statistical analysis

Since there was a clear drop in the number of red admirals captured around mid July we analysed early (up until 31 July, spring) and late (from 1 August, autumn) samples separately. We did not remove *δ*^2^H outliers in our analyses, since the large natural variation in *δ*^2^H makes it impossible to ensure that outliers (as long as they are within a possible biological range) are not recruits from other populations. SPSS 15.01 assigned only 7 *δ*^2^H values as “outliers” when looking at the data divided according to study year and season. Analyses were not affected by the presence or absence of the outliers. SPSS 15.01 was used for all statistical calculations.

We used Mann-Whitney U-tests to calculate differences in median passage dates between various subsets of the data. We used a t-test to analyse differences in *δ*^2^H between 2004 and 2005 in the autumn group. Differences in yearly mean date of passage during both seasons were analysed using a t-test. Differences in sex ratio between the two years as well as deviations from an equal ratio of males and females both in the whole data set and separately for spring and autumn were analysed using χ^2^-tests. Differences in mean date of capture for the different sexes in both seasons were compared using t-tests. To analyse if there was any difference in sex ratio between days with low and high migratory intensity, we divided the autumn material into two groups (the spring data was not analysed because of the low numbers of individuals available). This was done because we wanted to investigate if days with lower numbers were affected more by locally moving individuals (males) as compared to intense migration days where we expected both sexes being represented at equal numbers. For 2004, we designated days with more than ten individuals captured as high intensity days and for 2005 we lowered the limit to more than five since total numbers observed was smaller that year. Differences between the two groups were analysed using a χ^2^-test.

To analyse differences in lipid reserves we performed an ANOVA with lipid class as dependent variable and used sex, study year and sample season as fixed factors. We included all possible two-way interactions and removed them in a backward fashion. Since we had a significant interaction between season and sex we performed separate ANOVA tests for males and females.

The raw data used in all analyses is provided as Additional file 1.

## Results

### Timing and intensity of migration

#### Spring migration

**Fig. 3:**
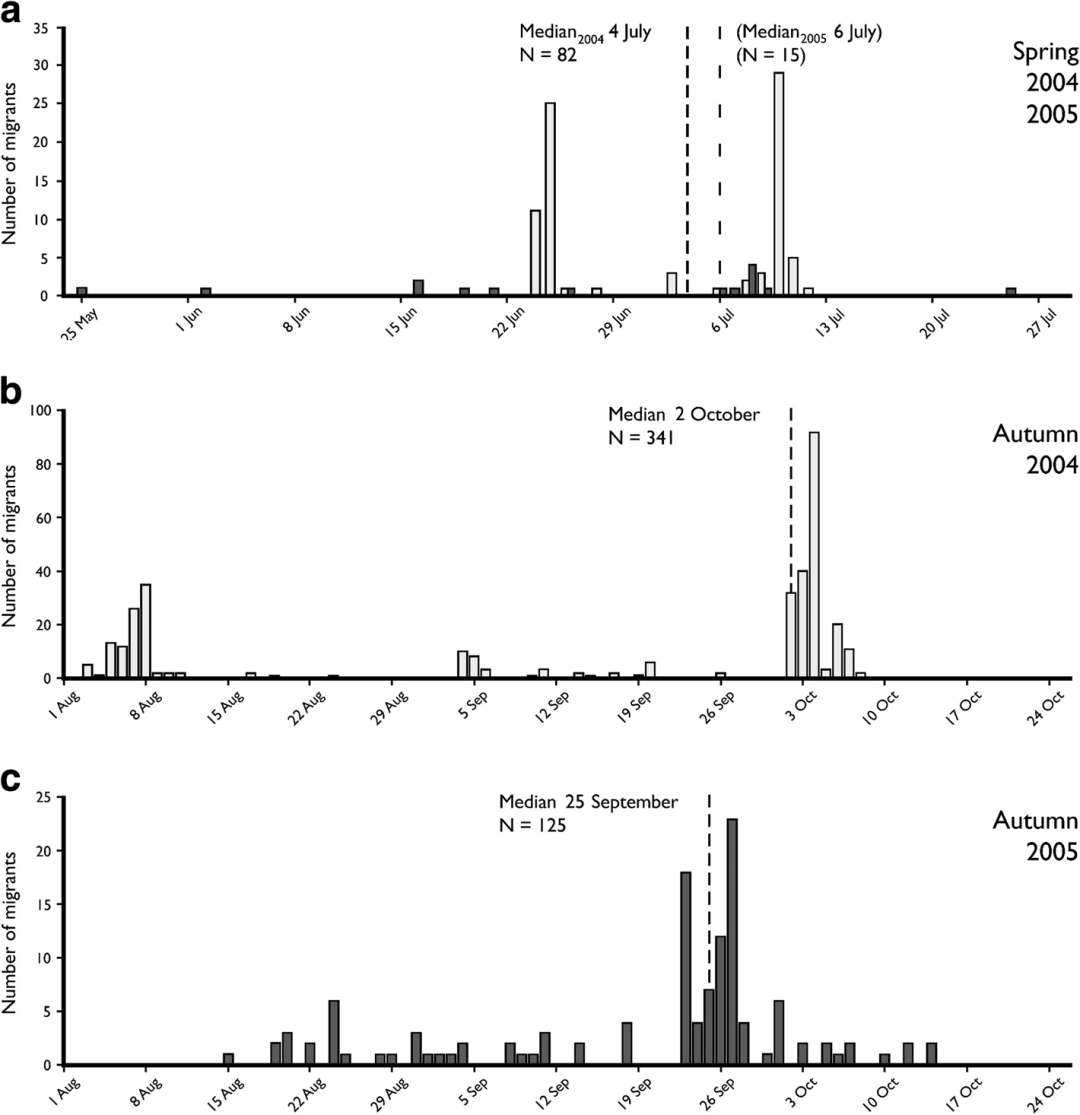
The number of red admirals caught per day in traps for two complete seasons (1 April to 1 November) 2004 and 2005. The captures are from **a** both spring seasons, **b** autumn 2004, and **c** autumn 2005. Note the different scale on the y-axis. The highest number of red admirals were captured in autumn compared to spring, and 2005 had much higher numbers than 2004

The total number of red admirals captured during spring migration was 82 in 2004 and 15 in 2005. The median date of passage for the spring migration was July 4 in 2004 (range: 24 June – 12 July) and July 6 in 2005 (range: 25 May – 25 July) (Fig. 3a). Since we had very few individuals in 2005 this value may be somewhat unreliable, although it was very close to the passage date noted the previous year. The median date of passage for the autumn migration was October 2 in 2004 (range: 3 August – 8 October, *N* = 341) (Fig. 3b) and September 25 in 2005 (range: 15 August – 14 October, *N* = 125) (Fig. 3c). There was no significant difference between years for the median passage dates in spring (Mann-Whitney U-test, U_95_ = 419.5, Z = 1.95, N_2004_ = 82, N_2005_ = 15, *p* = 0.051). The median dates of passage in the autumn was however significantly different between years (Mann-Whitney U-test, U_464_ = 18,392, Z = 2.27, N_2004_ = 341, N_2005_ = 125, *p* = 0.023).

### Measured and predicted *δ*^2^H values

#### Rybachy

**Fig. 4:**
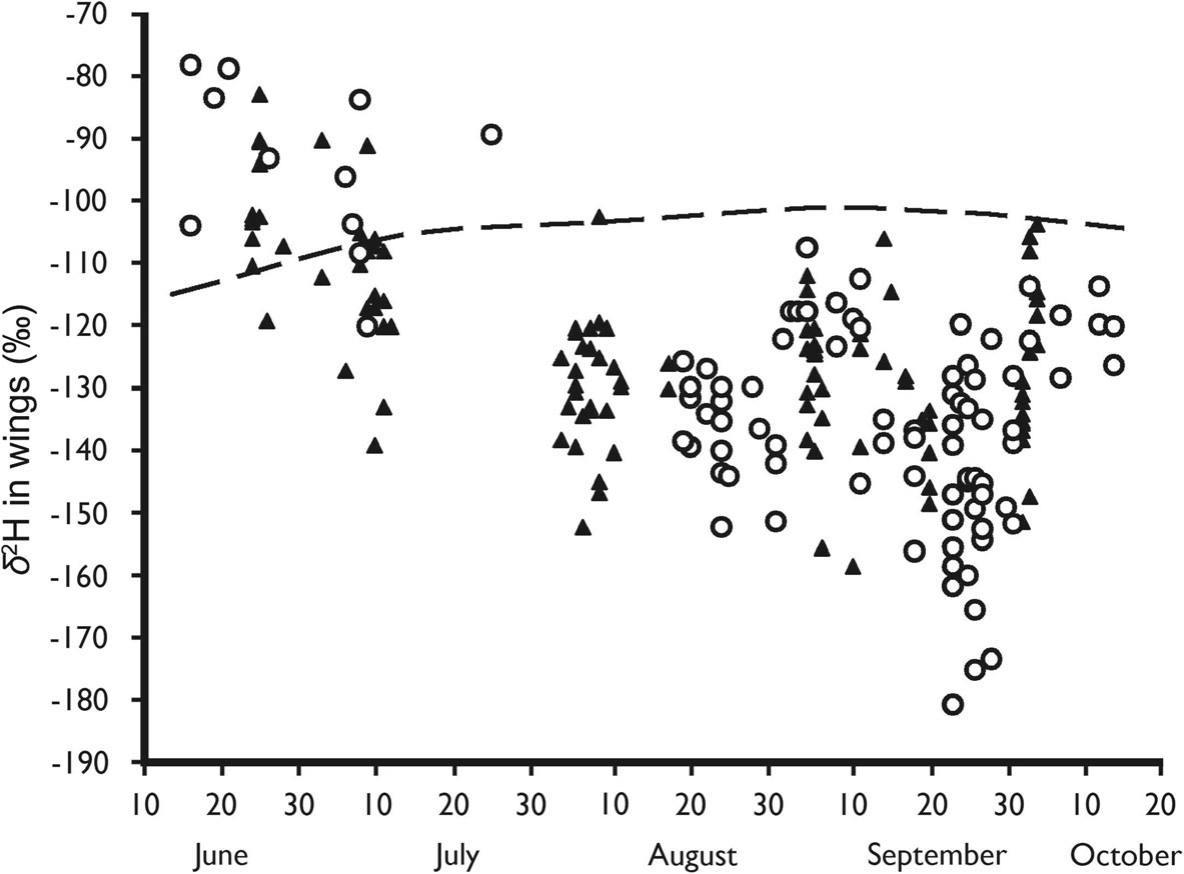
Measured *δ*^2^H values in the wings of red admirals sampled from the trap captures at Rybachy in 2004 (▲) and 2005 (○). The broken line shows the expected value for locally hatched individuals from the Rybachy region. For details on calculation of this estimate see text and Brattström et al. [14]

The *δ*^2^H values differed between the spring and autumn individuals for both years (Fig. 4). The expected *δ*^2^H values for locally grown butterflies (for details see [14]) show that all individuals from the autumn samples were of either local or from more north-eastern natal origins. The spring individuals in 2004, especially those sampled later in that period corresponded with local and more southerly origins. In 2005, all spring individuals had *δ*^2^H values corresponding to more southerly origins. The mean *δ*^2^H values measured during autumn was − 129.7 ‰ ± 1.39 ‰ (SE) in 2004 and − 136.3 ‰ ± 1.82 ‰ (SE) in 2005 and this difference was significant (t-test, *t* = 2.86, N_2004_ = 86, N_2005_ = 79, *p* = 0.005).

#### Estonia and Poland

**Fig. 5:**
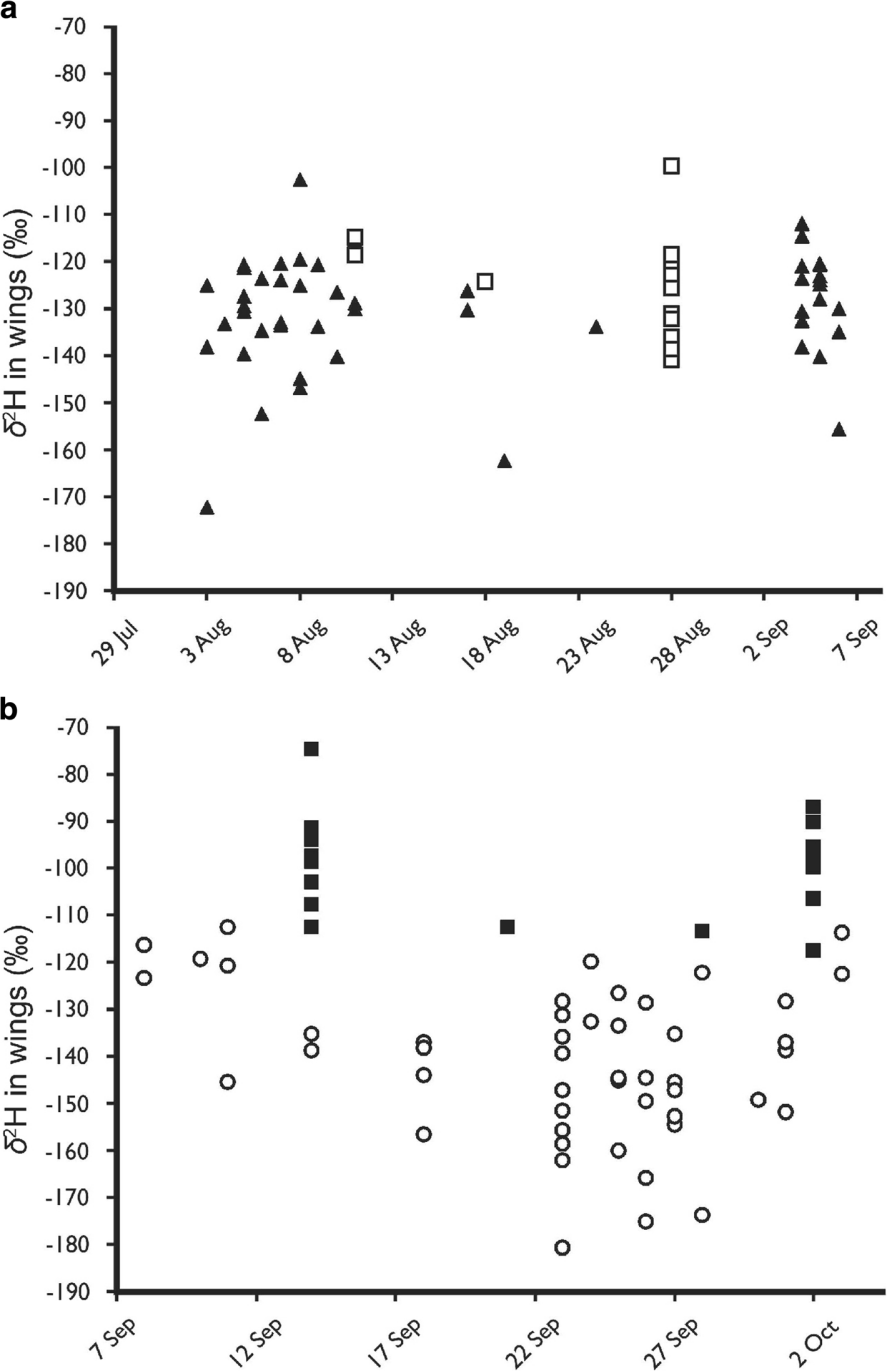
Measured *δ*^2^H values in the wings of red admirals sampled in **a** Estonia (□) in 2004 and **b** Poland (■) in 2005. Values are plotted together with the Rybachy values from the same time periods

The *δ*^2^H values in samples from our two inland reference locations are shown in Fig. 5, plotted in relation to the samples from Rybachy captured at a similar time. The range of *δ*^2^H values is far greater in the Rybachy samples (2004: min = − 172.1 ‰, max = − 102.4 ‰, range = 69.7 ‰, and 2005: min = − 180.8 ‰, max = − 107.3 ‰, range = 73.5 ‰) than for the inland locations (Estonia, 2004: min = − 140.8 ‰, max = 99.4 ‰, range = 41.4 ‰ and Poland, 2005: min = − 117.3 ‰, max = − 74.4 ‰, range = 42.9 ‰.

### Differences in sex ratios

There was no difference in median date of capture between sexes during spring (Mann-Whitney U-test, U_42_ = 237.5, Z = 0.082, N_Males_ = 23, N_Females_ = 21, *p* = 0.94) or autumn (Mann-Whitney U-test, U_162_ = 3043.5, Z = 0.73, N_Males_ = 96, N_Females_ = 68, *p* = 0.46), nor was there any difference in sex ratio depending on the intensity of the migration (χ^2^_1, 165_ = 0.78, *p* = 0.38). There was no difference in sex ratio between the two years (χ^2^_1, 208_ = 0.21, *p* = 0.89) and the pooled data from both years showed a sex ratio that was significantly biased towards more males (χ^2^_1, 208_ = 4.33, *p* = 0.038). When analysing the spring and autumn material separately, it was clear that this difference was only present in the autumn (spring: χ^2^_1, 43_ = 0.23, *p* = 0.88; autumn: χ^2^_1, 165_ = 5.10, *p* = 0.024).

### Breeding status of females

All of the females captured during the spring season had developing eggs in the abdomen (N_2004_ = 16 (Date: 24 June – 12 July), N_2005_ = 5 (16 June – 8 July). During the late summer/autumn season we found no visible eggs in any of the sampled females (N_2004_ = 35 (Date: 3 August – 4 October), N_2005_ = 33 (19 August – 14 October)).

### Differences in lipid content

**Fig. 6:**
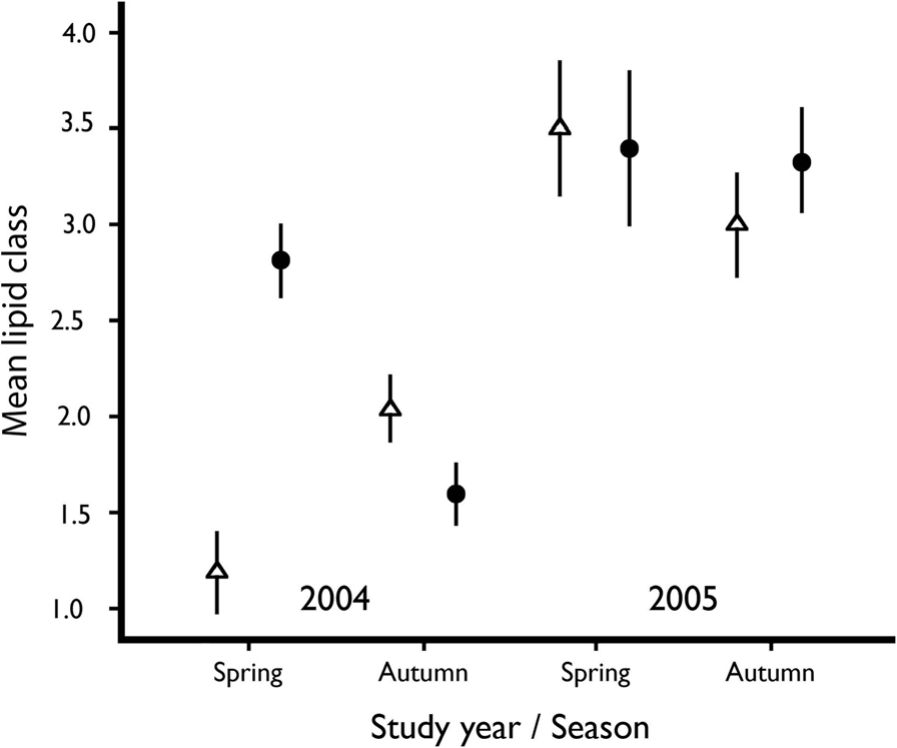
Mean lipid class assigned to male (∆) and female (●) red admirals captured at Rybachy in two different seasons 2004 and 2005. The difference between the years was significant and there was also a significant effect from season in the females. The errorbars represent ±1 Standard Error

The ANOVA analyses on visually estimated lipid classes showed a significant effect of year for males (Table 2a), and for females we found significant effects of both year and season (Table 2b). Both sexes were assigned to higher lipid classes in 2005, but for females there was less effect when comparing only spring samples (Fig. 6).

**Table 2:** Results of the ANOVA test of effects from study year and sample season on the assigned lipid class for males and female red admirals (*Vanessa atalanta*) sampled in spring and late summer/autumn in 2004 and 2005 at Rybachy, Kaliningrad. The presented results are the final model that remains after the non-significant interaction between the two factors have been removed

Variable	S.S.	df	*F*	*P*
a) Males				
Sample season	3.4	1	1.64	0.202
Study Year	38.9	1	18.76	< 0.001
Error	240.6	116		
Total	956.0	119		
b) Females				
Sample season	12.1	1	8.41	0.005
Study Year	48.3	1	33.6	< 0.001
Error	123.5	86		
Total	760.0	89

## Discussion

It is clear from our study that the migrating red admirals that pass the coastal site at the Curonian spit do so during two distinct periods, with a clear drop in migratory activity during mid summer. The median capture date was similar over the two studied years for spring and autumn. Even though there was a significant difference between the times of autumn passage, the absolute difference was not more than a week, suggesting a regular migration pattern between years.

The H isotope composition of precipitation correlates with geographic location, decreasing in *δ* ^2^H from south-west to north-east in Europe [11, 29, 42] and such patterns are reflected in foodwebs. So, this distinctive isotopic gradient can be used to infer origin over relatively limited geographical regions as shown for a sedentary butterfly species in Sweden [16]. This means *δ*^2^H can be used to help define the natal origin of red admirals from Europe over larger geospatial range, which has also been confirmed in a European wide study of red admirals [13]. The methods have also recently been used to understand the migration of pained ladies [50]. We found distinct differences in admiral *δ*^2^H values between the spring and autumn seasons at Rybachy, and therefore presumably their areas of natal origin. The range of *δ*^2^H was also wider at Rybachy (− 181 to − 78) as compared to Estonia and Poland (− 174 to − 100), suggesting a larger variation of origin at the coastal site compared to the inland sites. In spring, every female captured had developing eggs in the abdomen, but during autumn not a single female with eggs was found. We also found an unexpectedly skewed sex ratio in favour of males, but only during autumn. Difference in lipid content was also present, but this difference was more pronounced when comparing years than seasons. In 2005, red admirals carried considerably larger lipid reserves than the preceding year, suggesting more favourable conditions for pre-migration fuelling and possibly shorter migration.

### Timing of migration

In a previous study in Denmark, the median date of observed migration of red admirals over six years (1995–2000) was September 26. At this site in Denmark the earliest date with observed migration was August 13, and the latest October 30 [28]. Thus, the difference between earliest and latest mean date of migration was more than six weeks. At Falsterbo in southern Sweden the median passage of red admirals was studied by visual counts of migrating butterflies between 2004 and 2006 [15], and showed median dates (2004: September 6, 2005: August 31 and 2006: September 24) somewhat earlier than in Denmark. The median dates of our captured individuals at Rybachy were similar to other reported passage dates from northern Europe, but since we only have data from two seasons we cannot say if variation at this location is as large as observed in Denmark [28] and in southern Sweden [15]. It is clear from the different studies of red admiral migration that variation in many parameters of butterfly migration (for example timing and origin of migrants) can be substantial, and likely heavily dependent on yearly differences in weather, winds and reproductive success (e.g. [14, 15, 28, 36]). Data from reported sightings [28], standardized counts [15] and passive trap captures (this study), all result in similar time periods. It is therefore reasonable to assume that the main red admiral autumn migration in northern Europe occurs around September in most years. However, the difference in timing and fuel content may reflect a difference in migration distance between years, where the red admirals may reproduce at different latitudes in different years. Future studies, we suggest, would benefit from more standardized counting to make comparative studies easier [21].

When looking at the pattern of captures over the season, it was evident that red admirals do not pass Rybachy continuously, or within one single migration period. Instead, many days may pass without a single captured individual, followed by peaks in the capture data when half of the sampled individuals from one year were captured over the course of a single week (Fig. 3). This pattern was especially pronounced during autumn. A similar temporal pattern of red admiral migration has been reported at Falsterbo in southern Sweden recorded by visual observations [15]. We may ask what is the reason for these dramatic differences in migratory intensity that we found? Studies of the effect of weather on migration of red admirals at coastal sites have shown that wind direction is important to initiate large-scale migration [15, 36]. The above-mentioned studies were performed at locations where red admirals are about to cross open water and therefore may be more dependent on favourable winds than at inland sites. Such wind sensitive departures have been shown for migrating songbirds at coastal sites (e.g. [3], see also [6, 44, 45, 56]).

During migrations and in situations with winds from the east, red admirals may concentrate in large numbers at the coastal site at Rybachy. Especially high numbers could be the result of large-scale hatching events, occurring after cold periods and initiated by warm weather. Both explanations are weather dependant, but in different ways, with the first affected by wind speed and direction during migration and the second affected by temperatures at development. In our study, both factors may be involved in the migration pattern observed.

### Skewed sex ratios

In autumn of both years, we found more males than females in our samples from the Rybachy migration site. In many butterflies, males hatch before the females [25], but we found no difference in mean date of capture for the two sexes of red admirals during autumn migration at Rybachy. Theoretical modelling of protandry suggest that butterfly males in species that hibernate before reproduction should hatch before females [55], and this development pattern would also most likely be selected for in migratory species. In butterflies, often more males than females are encountered in the field, but the actual sex ratio as determined by observing hatching individuals is 1:1 [1]. A suggested reason for this is that the males move around more than females, and therefore are observed more often [1]. Brattström [12] found skewed sex ratios when sampling red admirals in Italy during late autumn and early spring where the red admirals are breeding. However, this skewed male sex ratio was interpreted as a sexual difference in hill-topping behaviour [18], at the high elevation capture site [12]. When skewed sex ratios that are not just artefacts of sampling techniques [1, 12] are encountered in butterflies and moths, these are almost exclusively female-biased and are caused by large scale infection of male-killing *Wolbachia* parasites (e.g. [32, 54]). In at least one butterfly species, a system exist were a male-biased sex ratio is the norm [51], but it seems unlikely in our study.

Sex and age differences in migration distance and wintering areas is common for migratory birds (e.g. [5, 7, 26, 33, 34, 37]). It is, however, questionable if sexual difference in wintering areas are a reasonable explanation for the sex ratio we found among the red admirals at our study site. We found developing eggs in all females captured in spring, but not in females during autumn. This shows that red admirals hatched in northeastern Europe and passing Rybachy, will most likely not reproduce in this region but migrate south before reproducing. The higher fat levels in late autumn individuals, especially in 2005 also suggest migration disposition and preparation for long-distance movements. Migrating insects, in general, show less difference between sexes during the migratory flight period than during reproduction. After the flight phase, changes in juvenile hormone levels reshape the insects from migratory to breeding states were sexual differentiation are more marked (for review, see [24]). Since we know that red admirals migrate to the Mediterranean area to reproduce during winter, males and females must share wintering areas in this region [20, 47]. Mating before migration is unlikely in red admirals, knowing that migration and subsequent breeding are two distinctly different phases in the life cycle of migratory insects [22]. We may therefore ask if the sexes in a sub-population of winter-hibernating red admirals use different winter regions and is reflected in our data from Rybachy? We believe that males captured at Rybachy do not visit the region for hibernation, but must instead be captured on migrating passage to reach more suitable winter habitat for reproduction (e.g. [20, 47]). Experimental studies of hibernating red admirals show that mortality rates increased dramatically in a moist environment [35], suggesting Rybachy is likely bad for hibernation. The observation period for this study, however, was just two weeks. Future studies need to reveal if skewed sex ratios exist at other locations for migrating insects present in the northern range of their distribution.

### Differences in lipid content between years and seasons

Even though it has been known for a long time that lipid content can vary extensively in migrating monarch butterflies (*Danaus plexippus*) [9], and that red admirals can store large amount of lipids [27], the difference in lipid content between years found in this study was unexpected. We also observed a higher number of individuals in 2004, indicating that it was a year with more successful breeding in the northern part of the range. The high numbers cannot be due to a second influx from the south later in the season, since most individuals found during autumn showed *δ*^2^H values of local or more northern origin. One possibility is that the large number of red admirals produced during 2004 meant that food sources were limited in the source area, and individuals therefore may be leaner than in 2005. Another possibility is that the foraging situation prior to capture at Rybachy was more favourable in 2005 compared to 2004, enabling red admirals to put on larger fuel stores before reaching the capture site. A study from Denmark suggested that red admirals in northern Europe can die in large numbers from starvation if weather conditions during autumn limit the available food and time for feeding [27]. The red admirals in 2004 could therefore have been under time pressure and forced to leave the northern regions with lower fat reserves (for discussion of time-minimized migration and departure fat loads, see [8]), but this remains to be shown. Red admirals captured in autumn 2005 had lower mean *δ*^2^H values than in 2004, suggesting a more northerly origin than 2004. Thus, it could be that in 2004, we primarily sampled locally hatched and not migratory individuals, but in 2005 we captured a majority of migrants with lipid stored ready for long distance flights. Plotting the fat scores onto the data shown in Fig. 3 suggest that the fat scores change towards the end of 2005 with individuals having higher lipid scores by the end of the migratory period (Additional file 2: Figure S1).

If fat stores found in the migrating red admirals are important for reproduction, we would expect them to be at their peak prior to reproduction in spring [49], and in our samples from 2004 we see that females, even though they were leaner than in 2005, have larger lipid reserves than the males. A study from Italy comparing fat content between autumn (before reproduction) and spring (before migration) found no difference between these two groups [12], but that study included only males. At this point, we cannot fully resolve this question with our data but the presence of differences in lipid content between years, and also to some extent between sexes, is something that deserves more attention in future studies.

### Effects of coastal location

We found a wider range of *δ*^2^H values in the Rybachy red admiral samples than samples from the inland locations of Estonia and Poland. This qualitatively suggests that red admirals at this coastal site arrive from a diverse catchment area, while the majority of individuals sampled at inland locations were from the local surrounding areas. Hansen [28] reported higher numbers of red admirals from coastal locations in Denmark compared to inland locations during autumn migration. Studies of flight behaviour of red admirals [10, 41], as well as monarchs [43] in coastal regions show that butterflies tend to follow coastlines and avoid flying over open water. This behaviour leads to concentrations of individuals from diverse locations ending up at coastlines that roughly follow the migratory direction, just as is shown in our samples from Rybachy. Migration along coastlines have further been shown in songbirds to be used to compensate for wind drift [2], which may also be a benefit explored by migrating butterflies.

## Conclusions

There are few locations in Europe where it is possible to systematically sample migrating butterflies over multiple seasons and in relatively high numbers. Rybachy in Kaliningrad represents one such location, where our data clearly demonstrate that new insights on timing of migration, population source areas, and fat content of migrating individuals as well as sex ratios can be gained for migrating butterflies. Unfortunately, since no similar data sets, systematically recording migrating insects in Europe [15, 31], are available we cannot make comparisons with this or other species collected at other locations, as our study represent a first attempt to fill this information gap. Coastal sites with channelling topography concentrate migrating butterflies, and will enable sufficient sampling with passive traps. We therefore hope that our study will inspire bird observatories over Europe and elsewhere, to include studies of migrating insects such as day-migrating butterflies in their monitoring programs.

## Additional files

Additional file 1: Raw data on date of capture, sex, presence of eggs, fat class and deuterium for red admirals captured at different study sites. (XLSX 19 kb)

Additional file 2: Graphs show the same deuterium data as Fig. 3 in the main text, but with the two years plotted separately. The fat scores are shown as different colours. The fat scores are shown as different colours. (PDF 257 kb)

## Abbreviation

VSMOW-SLAP: Vienna Standard Mean Ocean Water – Standard Light Antarctic Precipitation
