# Supplementary material for: Geographic origin and migration phenology of European red admirals (Vanessa atalanta) as revealed by stable isotopes
Source: Mov Ecol. 2018 Dec 21;6:25. doi: 10.1186/s40462-018-0143-3 (PMC6302385; doi:10.1186/s40462-018-0143-3)

Supplemental Figure 1:

Graphs show the same deuterium data as Figure 3 in the main text, but with the two years plotted separatedly. The fat scores are shown as differnet colours.

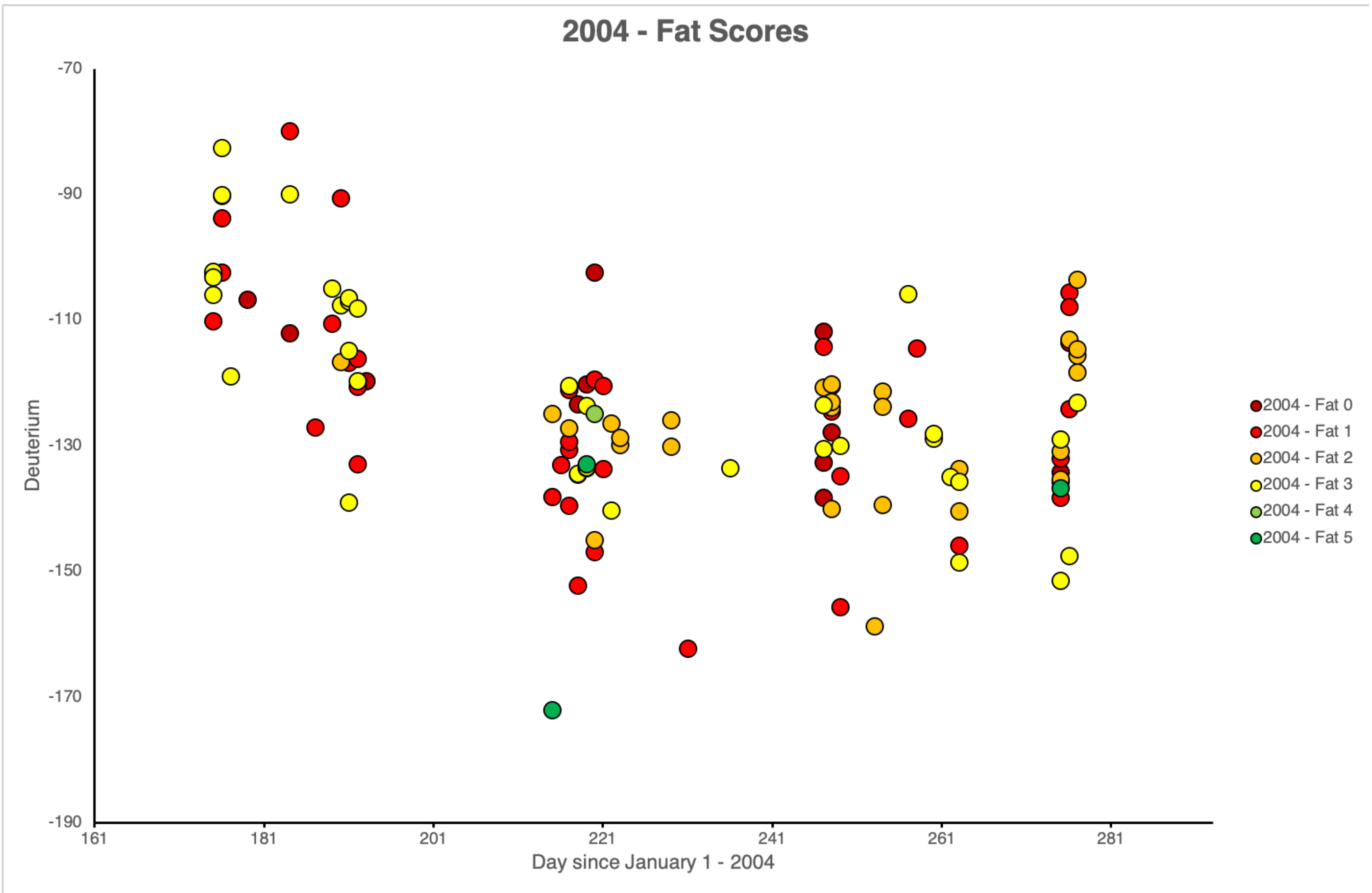

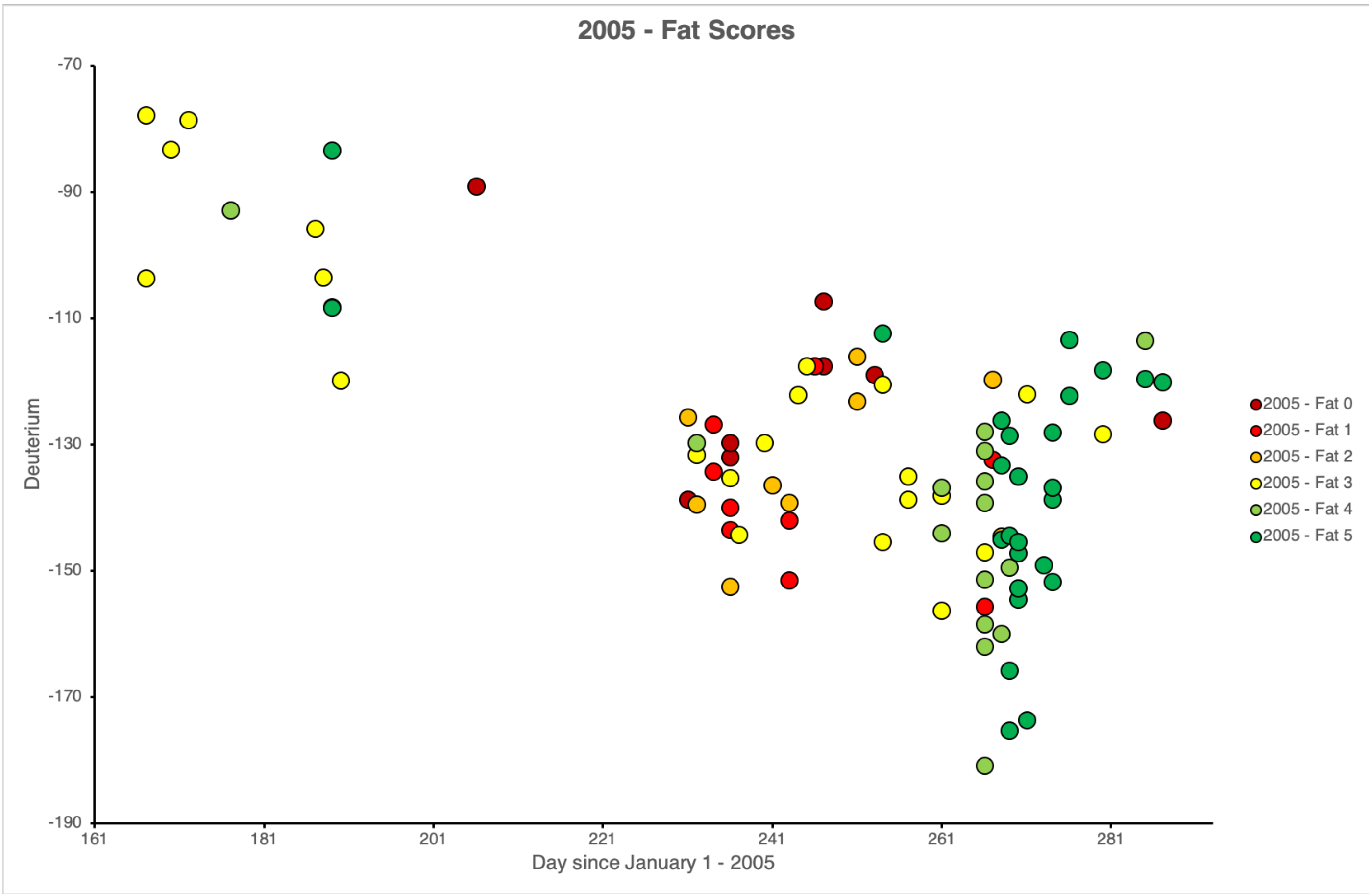

Supplement: Supplementary file 2 — Graphs show the same deuterium data as Fig. 3 in the main text, but with the two years plotted separately. The fat scores are shown as different colours. The fat scores are shown as different colours. (PDF 257 kb) [file 40462_2018_143_MOESM2_ESM.pdf]
